# Pills, PrEP, and Pals: Adherence, Stigma, Resilience, Faith and the Need to Connect Among Minority Women With HIV/AIDS in a US HIV Epicenter

**DOI:** 10.3389/fpubh.2021.667331

**Published:** 2021-06-21

**Authors:** Lunthita M. Duthely, Alex P. Sanchez-Covarrubias, Megan R. Brown, Tanya E. Thomas, Emily K. Montgomerie, Sannisha Dale, Steven A. Safren, JoNell E. Potter

**Affiliations:** ^1^Obstetrics, Gynecology and Reproductive Sciences, Division of Research and Special Projects, University of Miami Miller School of Medicine, Miami, FL, United States; ^2^Obstetrics, Gynecology and Reproductive Sciences, Division of Gynecologic Oncology, University of Miami Miller School of Medicine, Miami, FL, United States; ^3^Medical Education, University of Miami Miller School of Medicine, Miami, FL, United States; ^4^Miami Center for AIDS Research, University of Miami Miller School of Medicine, Miami, FL, United States; ^5^Department of Psychology, University of Miami, Coral Gables, FL, United States

**Keywords:** women and HIV, adherence, minority health and mental health, mobile health, mixed methods

## Abstract

**Background:** Ending HIV/AIDS in the United States requires tailored interventions. This study is part of a larger investigation to design mCARES, a mobile technology-based, adherence intervention for ethnic minority women with HIV (MWH).

**Objective:** To understand barriers and facilitators of care adherence (treatment and appointment) for ethnic MWH; examine the relationship between these factors across three ethnic groups; and, explore the role of mobile technologies in care adherence.

**Methods:** Cross-sectional, mixed-methods data were collected from a cohort of African-American, Hispanic-American and Haitian-American participants. Qualitative data were collected through a focus group (*n* = 8) to assess barriers and facilitators to care adherence. Quantitative data (*n* = 48) surveyed women on depressive symptomology (PHQ-9), HIV-related stigma (HSS) and resiliency (CD-RISC25). We examined the relationships between these factors and adherence to treatment and care and across groups.

**Findings:** Qualitative analyses revealed that barriers to treatment and appointment adherence were caregiver-related stressors (25%) and structural issues (25%); routinization (30%) and religion/spirituality (30%) promoted adherence to treatment and care. Caregiver role was both a hindrance (25%) and promoter (20%) of adherence to treatment and appointments. Quantitatively, HIV-related stigma differed by ethnic group; Haitian-Americans endorsed the highest levels while African-Americans endorsed the lowest. Depression correlated to stigma (*R* = 0.534; *p* < 0.001) and resiliency (*R* = −0.486; *p* < 0.001). Across ethnic groups, higher depressive symptomology and stigma were related to viral non-suppression (*p* < 0.05)—a treatment adherence marker; higher resiliency was related to viral suppression. Among Hispanic-Americans, viral non-suppression was related to depression (*p* < 0.05), and among African-Americans, viral suppression was related to increased resiliency (*p* < 0.04).

**Conclusion:** Multiple interrelated barriers to adherence were identified. These findings on ethnic group-specific differences underscore the importance of implementing culturally-competent interventions. While privacy and confidentiality were of concern, participants suggested additional intervention features and endorsed the use of mCARES as a strategy to improve adherence to treatment and appointments.

## Introduction

The United States (US) “Ending the Epidemic” initiative seeks to reduce new human immunodeficiency virus (HIV) infections by concentrating efforts to regions and subpopulations at greatest risk of acquiring the virus, as well as improving care for those already living with HIV/AIDS (1). The Centers for Disease Control (CDC) estimates that 65% of people with HIV (PWH) in US who know their status received some HIV care, whereas 54% were not HIV virologically suppressed and are more likely to transmit the virus to others (1). More than two-thirds (69%) of new infections in the US are attributed to people who are aware of their HIV positivity status, but do not receive treatment for their HIV. Ethnic minorities (minorities) and people living in the southern US are population subgroups more likely to acquire HIV (2). The southern region of Florida is considered a “hotspot” (1), with metropolitan Miami recording the highest rate of new HIV infections across all regions in the US (3). Adherence to HIV care and HIV anti-retroviral (ARV) regimens are public health priorities, not only for PWH, but also for persons at those at the highest risk of acquiring the infection. Adherence to ARV and to HIV care (appointments) is complex due to multi-faceted and interrelated barriers. Adherence to HIV care in the US overall is estimated to be around 71% whereas adherence among minority women is estimated to be 45–64% (4, 5). Among minority women, mental health diagnoses and HIV stigma are known factors that often co-occur; resiliency, social support and faith/spirituality facilitate optimal health care (4). To address challenges to adherence faced by minority women in southern Florida, a concurrent, pilot randomized controlled trial is investigating a mobile health intervention to improve outcomes for minority women with HIV (MWH), attending a large urban clinic in metropolitan Miami.

The current study uses a mixed method approach, through focus groups (qualitative) and survey (quantitative) analyses, to inform a future mobile technology (mHealth) intervention which aims to improve adherence to HIV care (appointments and ARV) (6–11). Most mHealth interventions consist of SMS/texting alerts and messaging or native smartphone apps; the majority are designed for international markets and have proved efficacious along the continuum of HIV care—from HIV prevention to HIV treatment (6, 11–14). A much smaller proportion supported women—designed typically for pregnant women and their newborns (10, 11). It is more common to find mHealth interventions tailored for adolescents and men who have sex with men (MSM) (15). Culturally tailored approaches that address multiple factors related to HIV-related stigma (16) and behavior change are needed (17). Another important consideration is tailoring mobile applications to attend to the needs of vulnerable populations (18), while addressing their privacy and confidentiality concerns (15).

The current study represents the formative work completed to inform the design of the mCARES texting system for MWH. The participatory process involved a mixed-methods approach, where quantitative surveys, medical information and qualitative data, in the form of focus groups, were gathered to understand the individual barriers and facilitators to treatment adherence. The objective is to understand the relationship between individual-level factors and HIV care adherence (ARV adherence and viral suppression), across different cultural minority groups, and the role of mobile technologies to support adherence. The study also explored participants' perceptions regarding the challenges posed by mobile technologies.

## Methods

### Study Design

This is a concurrent, mixed-methods study that began with the quantitative component in March 2019. The first round of focus groups took place in June 2019. This study describes data collected to inform the design and refinement of the mCARES (mHealth) prototype.

### Participants, Study Setting and Period

Women enrolled in HIV care with a recent history (12 months) of non-adherence were eligible for enrollment. The study site was a single-site facility that provides primary care and prenatal care to the largest group of ethnic minority African-American, Caribbean-American and Hispanic-American women living with HIV in southern Florida. Data collection took place from March 2019 – February 2020 at a Medical Center consisting of a large, public clinic, teaching hospital, and an academic research institution. Participants were compensated a maximum of $50 (given in the form of grocery store gift cards) to complete the surveys and participate in the focus group.

### Eligibility Criteria

Described in detail elsewhere (19), eligible women for both the quantitative and qualitative components of this study had either clinically confirmed detectable HIV (viral load) VL or missed visits, or self-reported not taking ARV as prescribed (i.e., missing doses or declining to take ARV).

### Texting System Prototype

The messaging system, mCARES (mobile communications for adherence reminders education and support), is a limited two-way texting system designed to send and receive simple text messages. To accommodate for the potential burden of accessing an “app” on a smartphone [i.e., smartphone ownership and fees associated with reliable internet access (19)], the texting system was programmed as a short messaging system (SMS) that runs on basic cellular phones—given the limitation that basic cellular phones, i.e., flip phones, do not support higher level MMS (multimedia messaging) signals. Another feature of mCARES is the customizable timing and frequency of the messages, to reduce the possibility of messaging or reminder fatigue (19).

### Qualitative Data

As part of a participatory process to inform the development of the mCARES intervention, we conducted a 90-minutes (approximately) focus group that involved patients registered in the clinic, with a recent history (12 months) of non-adherence.

Focus group questions explored: (a) barriers to- and facilitators of- care adherence; (b) personal factors that facilitated or impeded adherence to care (c) how reminders, alerts and psychoeducational material could facilitate care; (d) the role of texting to deliver the reminder, alerts and psychoeducational messaging; and (e) potential barriers and facilitators of mobile technology to support adherence to care.

Focus group participants were asked questions such as “What are the personal challenges to keeping appointments?,” “and for taking medications regularly?,” “How does a woman's state of mind, emotions, thoughts and mental health status play into why they miss appointments or don't take medications regularly?”

Using an iterative process, the audio recording was first transcribed verbatim, independently, by two study team members (APS, MRB), which was facilitated with a speech recognition software (20). A third study team member (LMD) reviewed the transcripts to resolve discrepancies. The data were transferred to a qualitative/mixed-methods data management and analysis software (21). The initial machine-generated data instances were then reviewed for relevancy. Instances were then consolidated and promoted to major themes.

### Quantitative Data

Baseline data were collected from both study arms. All participants completed a study-created form to report their socio-demographic characteristics and validated questionnaires that assessed depressive symptomatology, HIV-related stigma, resiliency and data regarding adherence to ARV. Participants' recent clinical history was extracted from electronic medical records (EMR). (See Instrumentation for details).

### Instrumentation

#### Socio-Demographic and Clinical Data

Participants reported on their race, ethnicity, age, recent history of mental health and substance use. The clinic EMR was queried for participants' 12-month history of HIV viral load, clinic attendance, mental health conditions, psychiatric diagnoses and substance use.

#### Patient Health Questionnaire (Depressive Symptomology)

Depressive symptoms were measured using the Patient Health Questionnaire−9 (PHQ-9). Participants rated how often they have been affected by any of the symptoms stated (0 = “not at all” … 3 = “nearly every day”). The PHQ-9 is validated on a 3-point Likert-type scale (Cronbach's α = 0.89) (22).

#### HIV Stigma Scale

Internalized stigma was measured with the HIV–related stigma scale (HSS), a 40-item measure validated in English (Cronbach's α =0.96). Individuals rated statements such as “Some people act as if it's my fault I have HIV” on a 4-point Likert-type scale (1 = “strongly disagree” … 4 = “strongly agree”) (23).

#### Connor-Davidson Resiliency Scale

The Connor-Davidson Resiliency Scale (CDRISC-25), a validated 25-item questionnaire (Cronbach's α = 0.89), included statements such as “When things look hopeless, I don't give up.” Responses are on 5-point Likert-type scale (0 = “not true at all” … 4 = “true nearly all of the time”) (24).

#### Self-Report Measure for Medication Adherence

Medication adherence was measured using the Self-report Measure for Medication Adherence, which asks “Days Taken” over the last 30 days. This questionnaire was validated using an electronic drug monitoring device for HIV ARV (Cronbach's α = 0.84) (25).

## Results

### Qualitative Results

Results are specific to the focus group that took place among the English-speaking study participants. The majority were non-Hispanic Black (90%), ≤ 45 years-of-age (77%), unmarried (74%), and in primary care or gynecological care (70%). A total of eight individuals participated in the focus group.

### Barriers and Facilitators to HIV Care Adherence

From a total of 24 instances related to barriers to adherence, 7 themes were generated. From a total of 10 instances identified as promoters of adherence, 4 themes were generated (Table 1). The most salient themes identified as barriers to care were caregiver-related stressors (e.g., children or adult dependents) (25%), structural issues (e.g., clinic-related issues such as scheduling) (25%), and conscious choice (e.g., “no pills when drinking”) (21%). The most salient themes identified as promoters of adherence to care were routinization (e.g., always taking medications after meal) (30%), religion/spirituality (30%) (e.g., “turn to God” for support), and caregiver role (20%) (e.g., “be there for my kids”).

**Table 1:** Focus group qualitative results.

**Barriers to adherence (*N* = 8 Women; *N* = 24 instances)**	***n* (%)**
Caregiver role (ARVs and appointments)	6 (25)
Concurrent alcohol use (ARVs)	5 (21)
Schedule/Busy (ARVs and appointments)	3 (13)
Other circumstances (ARVs and appointments)	3 (13)
Structural (e.g., Clinic/Transportation): appointments	3 (12)
Medication side effects (ARVs)	2 (8)
Personal (e.g., forgetfulness, hiding medications) (ARVs)	2 (8)
Total	24 (100)
**Promoters of Adherence (*****N*** **= 10 instances)**	***n*** **(%)**
Caregiver role (i.e., responsibility for children)	2 (20)
Faith/Religion/Spirituality	3 (30)
Avoid medication side effects (i.e., take ARVs with meals)	3 (30)
Trust in medical personnel	2 (20)
Total	10 (100)

*ARV, anti-retroviral*.

### Role of Technology to Promote Adherence

The consensus amongst all focus group participants (*n* = 8; 100%) was that medication SMS/texting alerts and advanced appointment reminders would facilitate adherence. However, there was concern (*n* = 2; 25%) that an excessive number of alerts (“dings,” “buzzes”) would be bothersome. When explained that the system would allow for individual personalization for the frequency and timing of alerts, all participants (100%) expressed willingness to try the system. Participants (100%) agreed that educational and psychological/mental health messaging that would not compromise their privacy or HIV status could be supportive.

### Challenges Related to Technology

The focus group also explored potential challenges that may be introduced with the use of mobile technologies. All focus group participants (*n* = 8) expressed concerns regarding one or more aspects of privacy and confidentiality. Concerns included risk of unintended disclosure (to family or friends) and privacy of their data related to technology for the healthcare of persons with HIV. These concerns were raised by participants who had disclosed, as well as considerations for others who may not have disclosed their HIV status to others. Two participants (25%) relayed having been “outed” (publicly announced) on social media by relatives about their HIV status.

### Other Related Findings

#### Advice to Others

Nearly all focus group participants (*n* = 7; 88%) offered advice to the other group members on one or more items below. Advice was offered as a way to enhance the quality of the life of a woman living with HIV.

**Health-Related Advice**

- Adhere to other (non-HIV) medications, as well (e.g., medications for hypertension).
- Inquire from different sources about pre-exposure prophylaxis (PrEP) for HIV-negative partners.

**Advice to Support/Build Resiliency**

- Disclose HIV-positivity status to partners and family.
- Consider children and other dependents as motivators for staying healthy.
- Seek out ways to curb negative thoughts.
- Turn to spirituality/religion/faith, even if not religious, which is important (“look to God” for inspiration).
- Mental resiliency is needed (“it's a mind thing”).

#### Additional System Features

Nearly all focus group participants (*n* = 7; 88%) suggested additional features, which could also help improve adherence to care, including:

**Messaging to Build Resiliency and Support Adherence**

- Include messaging related to spirituality and faith (*n* = 5), but not religious messages (*n* = 1).
- Add messaging for other medical appointments and medications (*n* = 7).
- Peer group meetings, in addition to messaging, could provide additional support (*n* = 7).

#### Quantitative Results

Quantitative analyses were executed with SAS. The Wilcoxon Rank Sum procedure was used to test differences of depression (PHQ-9), HIV-related stigma (HSS), resilience (CD-RISC25), and days of ARV missed doses by demographic characteristics. The Spearman Coefficient tested the correlations between participant scores on each of the measures (i.e. PHQ-9, HSS, CD-RISC25), and the total ARV days missed. A *p*-value at a cutoff of 0.05 determined significance.

Data from all participants (*n* = 48) enrolled into the clinic's primary care, prenatal care or gynecological service with complete variables of interest were analyzed. As some participants were VL detectable and missed visits, we prioritized HIV detectability over missed visits, and 27% were VL detectable (>20 copies/mL) and 39% were “no-show” (i.e., did not cancel, did not call to reschedule before the end of the clinic day) for one or more of scheduled HIV-related appointments. The majority were non-Hispanic, African-American or Haitian-American (67%), >45 years-of-age (54%), not pregnant (84%). From the self-report measures for the Medication Adherence scale (Medication Adherence), 58% reported taking their ARV for all of the previous 30 days, 67% reported a frequency of “Always” taking their ARV, and 54% rated themselves as “Excellent” (Table 2).

**Table 2:** Demographic profile and baseline assessments by ethnicity, and correlation of assessments.

**Demographic characteristics**	**African-American (*n* = 20)** ***N* (%)**	**Haitian-American** **(*n* = 12)** ***N* (%)**	**Hispanic-American** **(*n* = 16)** ***N* (%)**	**Total (*n* = 48)** ***N* (%)**
**Age group**				
<45 years old	13 (65)	5 (41.7)	4 (25)	22 (46)
**Pregnancy status**				
Not pregnant	13 (65)	12 (100)	15 (94)	40 (84)
**Treatment adherence**	**African-American**	**Haitian-American**	**Hispanic-American**	**Total**
Viral load*				
Suppressed	14 (70)	8 (67)	13 (81)	35 (73)
Not suppressed	6 (30)	4 (33)	3 (19)	13 (27)
**Medication Adherence**				
Days taken = 30	13 (65)	7 (58)	8 (50)	28 (58)
Frequency = “Always”	13 (65)	9 (75)	10 (63)	32 (67)
Rating = “Excellent”	11 (55)	6 (50)	9 (56)	26 (54)
**Assessment**	**African-American** **median (IQR)**	**Haitian-American** **median (IQR)**	**Hispanic-American** **median (IQR)**	**Overall** **median (IQR)**
Depression (PHQ-9)	5 (5.5)	4 (9.5)	2.5 (7.5)	4.5 (7.5)
HIV Stigma (HSS)	91 (35.5)^a^	132 (28.5)^a^^,^^b^	102.5 (30)^b^	104.5 (42.5)^+^
Resiliency (CD-RISC25)	83 (24.5)	94 (13.5)	90.5 (35)	88.5 (26.5)
**Factors (spearman correlation)**	**PHQ-9**	**HSS**	**CD-RISC25**	
Depression (PHQ-9)	–	–	–	
HIV stigma (HSS)	0.534^++^	–	–	
Resiliency (CD-RISC25)	−0.486^++^	−0.057	–	
ARV missed: 30 days	0.163	0.166	−0.186	

+ *p < 0.01 (overall)*,

++ *p < 0.001*,

a *p < 0.05, comparison of African-American to Haitian-American*,

b *comparison of Haitian-American to Hispanic-American*.

*Patient Health Questionnaire = PHQ-9; HIV Stigma Scale = HSS; Connor-Davidson Resiliency Scale (25 items) = CD-RISC25*.

The distribution of scores was as follows: for depression (PHQ-9), the median was 4.5 (IQR = 7.5); for HIV stigma (HSS), the median was 104.5 (IQR = 42.5). The median resilience score (CD-RISC25) was 88.5 (IQR = 26.5) (Table 2).

We then measured the relationships between the different measures. Depression (PHQ-9) correlated positively to HIV-related stigma (HSS; *R* = 0.534; *p* < 0.001) and negatively to resiliency (CD-RISC25; *R* = −0.486; *p* < 0.001). Examined by viral suppression, higher depression and HIV-related stigma were related to viral load non-suppression; higher resiliency was related to viral suppression. For each comparison, the converse was also true—lower depression and HIV-related stigma were related to viral load suppression; lower resiliency was related to viral load non-suppression (Table 2).

We then measured the relationships between the different measures, across and within ethnic groups. We found significant differences for depression and viral suppression across the three ethnic groups (*p* < 0.05). *Post-hoc*, within-group comparisons for viral suppression revealed significant differences amongst the Hispanics for depression, where viral suppression was associated with lower depression (*p* < 0.05), and amongst the African-Americans for resiliency (*p* < 0.04), where viral suppression was associated with higher resiliency. For each comparison, the converse was also true (Table 3).

**Table 3:** Assessments by ethnicity and HIV viral suppression.

**Depression (PHQ-9)**	**African American (*n* = 20)**	**Haitian American (*n* = 12)**	**Hispanic American (*n* = 16)**	**Overall (*n* = 48)**
	**(median, IQR)**	**(median, IQR)**	**(median, IQR)**	**(median, IQR)**
VL suppressed	4.5 (4)	2.5 (11)	2 (6)	3 (6)
VL not suppressed	8.5 (15)	7.5 (7.5)	17 (19)	9 (13)^*^
**HIV Stigma (HSS)**				
VL suppressed	89.50 (27)	130 (31.5)	100 (29)^+^	100 (35)
VL not suppressed	108.50 (58)	140 (26)	111 (50)^+^	116 (21)
**Resiliency (CD-RISC25)**				
VL suppressed	86.5 (14)^#^	94 (19)	96 (29)	89 (22)
VL not suppressed	64.5 (41)^#^	92.5 (10)	54 (52)	77 (37)

* *p < 0.05 (overall)*,

+ *p < 0.05 (within Hispanic-American group, only)*,

# *p < 0.05 (within African-American group, only)*.

*VL, HIV viral load*.

## Discussion

Technology-based solutions geared toward to curbing the global HIV epidemic focused initially on social media, with a larger emphasis on prevention of HIV amongst high risk groups such as adolescents and men having sex with men (26); however, as more is learned regarding the multiple factors related to- and that impact- HIV behavior change, interventions are needed which are culturally tailored and take a psychoeducational approach to address the multi-level challenges persons living with HIV face (17). The COVID-19 pandemic has brought the world into a new era of healthcare delivery that must balance technological advancement—which has the potential to improve public health—with personalized approaches that afford equitable access for all population subgroups (27).

To maximize future uptake of a technology-based HIV intervention, we embarked on a mixed-methods investigation to inform the design of mCARES, a messaging and alerts system to improve adherence for a clinic population of ethnic MWH. The southern US states are a regional “hotspot” for new HIV infections (2). The state of Florida is not in the “deep south,” where health inequities are the most pronounced; nevertheless, with metropolitan Miami having the highest rate of new HIV infections across the US, interventions to support adherence are needed to help curb the epidemic (3).

### Qualitative Findings

The most salient themes that emerged were that caregiver-related stressors, structural issues and personal choice were the barriers participants identified, and that routinization, religion/spirituality and caregiver role promoted adherence to care. Our findings concurred with Fletcher et al. (28); study participants (59%) were US-based, Black non-Hispanic women, who reported on “hardiness,” spirituality/religion and family as strengths that helped them cope with their diagnoses. Similarly, Dale et al. (2018) (29) described that Black women with a history of trauma relied on resiliency and social support to overcome HIV-related stigma, which in turn promoted HIV adherence. In another study, Levison et al. (2017) (30) documented similar experiences among US-based LatinX immigrants, who reported that structural barriers such as institutional or personally enacted HIV stigma, structural issues (transportation, challenges with clinic) made adherence difficult while routinization (going to work every day) and religion/spirituality promoted adherence.

Focus group participants from the current study identified family as both a hindrance (25%) and a factor that facilitated adherence to care (20%). Furthermore, although the topic of disclosure was not probed, themes regarding disclosure to family and partners did emerge. In our study, participants relayed having been “outed” (publicly announced) about their HIV status by relatives, which was similar to a report of African-American women in US southern states (16). Participants concurred that texting—medication reminders, appointment alerts, psycho-educational messages (e.g., inspirational/religious/informational)—could facilitate adherence. Prior studies have reported positive outcomes from mHealth interventions (6–11). It is important to note, however, that metareviews have reported mixed results (6, 11–14). Another finding from our interviews is that participants voiced their privacy and confidentiality concerns, echoing our findings from a recent review of the literature (15).

### Quantitative Findings

We surveyed African-American, Haitian-American and Hispanic-American women, predominantly of Black race (76%). Our findings examining the relationships between the factors (i.e., mental health status, HIV-related stigma) that interfere with HIV care adherence (i.e., VL suppression) and factors (i.e., resiliency) that promote adherence were consistent with recent findings among other US cohorts of ethnic minority women (31) and among African-American women, specifically (32, 33). Comparisons across African-American, Haitian-American and Hispanic-American women with HIV have not been reported, however, the most relevant findings are discussed here.

Our findings are also consistent with what has been reported in the state of Florida, regarding significant correlations between HIV-related stigma (by healthcare workers) and ARV adherence (34), and HIV-related stigma and depression (35), although this is specific to a population of majority non-Hispanic Black men and women with HIV. Outside of the US, the relationship between HIV-related stigma and adherence has also been established among marginalized populations in the Dominican Republic, including men and women of Haitian descent (36). We did not find similar US-based studies, disaggregated by ethnicity, for comparison with other cohorts of Haitian women living in the US.

Among study participants in the current study, Haitian-Americans endorsed the highest level of stigma, followed by Hispanic-Americans; African-Americans endorsed the lowest level (*p* < 0.05). Our findings were similar to a study conducted in the Dominican Republic, where persons of Haitian descent, who are immigrants and have a history of discrimination in the Dominican Republic endorsed higher levels of stigma (36). In southern Florida, Hispanics are a minority-majority; Haitians are a minority. There is a history of stigmatization of persons of Haitian descent, who, in the early days of the HIV epidemic, were deemed a high-risk group. This could explain why the Haitian-American women in this study endorsed higher levels of stigma, compared to the Hispanic-American women.

Our quantitative findings were consistent also with recent studies reporting on factors that positively impact adherence to HIV care. Among US African-American women, resilience correlated significantly to medication adherence (33). Outside of the US, depression and resilience were correlated inversely, among South African women (99% of Black race) (37), and Latin American men and women (38).

Factors that influence adherence to HIV care are not only related to each other, but significant interactions (i.e., mediation and moderation) have been documented as well. It was beyond the scope of this study to conduct such analyses. It is worth noting, however, that a recent US-based study of predominantly low-income women, demonstrated in higher order analyses significant moderating effects of these relationships (i.e., depression, HIV-related stigma, ARV adherence) (28).

### Data Synthesis: Opportunities to Overcome Challenges

The evidence in the literature is growing, regarding the interplay between stigma experienced by marginalized populations and mental health conditions see (39). From the current study, quantitative assessments revealed significant relationships between adherence to ARV and to HIV viral load suppression and HIV-related stigma, depressive symptoms, and resilience. The following were identified from the current study's focus group as opportunities to overcome challenges that interfere with adherence.

### Pills

Participants were willing to engage with mHealth interventions as an additional strategy to support their adherence to anti-retroviral medications and appointments. They were also interested in additional features regarding medications prescribed for other chronic conditions. mHealth technology platforms, however, must guarantee privacy and confidentiality.

### PrEP

Participants wanted education about PrEP for HIV-negative partners. Those already experienced with- or who had prior knowledge of- PrEP, advised others in the group to seek out additional information on PrEP.

### Pals

Participants viewed personal relationships and support from family and friends as promoters of adherence.

### Faith

Spirituality/religiosity was endorsed as a promoter of mental resiliency and adherence. Participants suggested messaging geared toward spirituality, and advised each other to seek out spirituality, prayer or religious affiliation for strength.

### Resiliency

Mental resiliency (“it's a mind thing”) and curbing negative thoughts can help overcome challenges encountered by a person living with HIV and as a way to cope with others' opinions about their diagnosis (e.g., HIV-associated stigma).

### Need to Connect

Participants expressed a need for peer support, in the form of group meetings, as a way of sharing and exchanging experiences and knowledge with other women living with HIV.

### Study Limitations

The generalizability of study findings, both qualitative and quantitative, are limited by the small sample size of focus groups (*n* = 8) and cohort size (*n* = 48), and that participants represented a convenience sample of minority women living with HIV, receiving care from a single clinic site. Participants were enrolled in an ongoing study examining the feasibility of an mHealth intervention designed for multiple linguistic groups. The study is also limited by cross-sectional design and potential bias in self-reporting of data. The study did not account for other factors, such as length of time in the US for non-US born participants, another surrogate of acculturation.

Additional limitations exist in understanding the relationship between adherence to ARV (missed doses, self-report) and viral suppression (clinically confirmed). Not all women in our study are on the same ARV regimen. Individual ARVs have different half-lives (length of time in the body), and so missed doses may have differing effects on viral load. This study did not collect participants' ARV regimen; we, therefore, were not able to account for these differences. Despite these limitations, the data were a glimpse into the clinical profile of these women and the interplay of different factors that may be related to adherence to ARVs and to their clinical appointments.

## Conclusion

Among this cohort of ethnic minority women followed in a US safety net HIV clinic, the results regarding the complex nature of adherence were consistent with previous findings. Our cross-sectional data found these factors correlated to each other and were related to HIV care adherence, specifically HIV viral load suppression, and subjective assessments of HIV anti-retroviral adherence. To our knowledge, this is the first study to examine the relationship between HIV-related stigma, depression, resilience and adherence to HIV care (anti-retrovirals and appointments), among a cohort of African-American, Haitian-American and Hispanic-American women living with HIV in the US.

Synthesizing quantitative and qualitative results, we found that depression, HIV-related stigma and caregiver responsibilities were factors that impeded adherence to care; resilience, family, social support and faith/spirituality promoted adherence, among this diverse group of African-American, Hispanic-American and Haitian-American women. While we remain cognizant of the limitations of our sample (i.e., size, convenience), our findings support existing data on factors that impede and promote adherence and elucidate important considerations for this group of women living in the US, who represent different cultural groups. Another important finding not often cited in previous literature is that focus group participants also requested messaging related to spirituality/religion/faith, and participants proposed group meetings for additional education/health (e.g., PrEP) and emotional support. More importantly, the study provided a baseline understanding of the nuanced differences among a diverse group of ethnic minority women, receiving care in large public HIV clinic, and a way forward to incorporate these findings into the design of mCARES, a digitized intervention to support adherence to care.

Mobile technologies have the potential to supplement HIV care, and our focus group participants endorsed mHealth as a viable option to support adherence, as long as their privacy and confidentiality were not compromised. The COVID-19 pandemic catapulted the world into virtual healthcare, and so understanding how mobile technologies work for all will maximize uptake and use among those with the greatest need.

## Data Availability Statement

The datasets presented in this article are not readily available due to restrictions, as stated the Department of Health and Human Services. Requests to access the datasets should be directed to http://privacy.med.miami.edu/employees/data-use-agreements.

## Ethics Statement

The studies involving human participants were reviewed and approved by University of Miami Institutional Review Board (#20170287). The patients/participants provided their written informed consent to participate in this study.

## Author Contributions

LD: conceptualized the original protocol and study and produced the first manuscript draft. LD, AS-C, MB and TT participated in data collection. LD and AS-C performed the data analysis. EM and JP: provided reviews and editorial comments. SD and SS provided input to the original protocol design and manuscript. All authors contributed to the article and approved the submitted version.

## Conflict of Interest

The authors declare that the research was conducted in the absence of any commercial or financial relationships that could be construed as a potential conflict of interest.
